# Retraction: Experimental and theoretical studies of the nanostructured {Fe_3_O_4_@SiO_2_@(CH_2_)_3_Im}C(CN)_3_ catalyst for 2-amino-3-cyanopyridine preparation *via* an anomeric based oxidation

**DOI:** 10.1039/d2ra90093a

**Published:** 2022-09-23

**Authors:** Mohammad Ali Zolfigol, Mahya Kiafar, Meysam Yarie, Avat (Arman) Taherpour, Mahdi Saeidi-Rad

**Affiliations:** Department of Organic Chemistry, Faculty of Chemistry, Bu-Ali Sina University Hamedan Iran zolfi@basu.ac.ir mzolfigol@yahoo.com +98 8138257407 +98 8138282807; Department of Organic Chemistry, Razi University P.O. Box: 67149-67346 Kermanshah Iran; Medical Biology Research Center, Kermanshah University of Medical Sciences Kermanshah Iran

## Abstract

Retraction of ‘Experimental and theoretical studies of the nanostructured {Fe_3_O_4_@SiO_2_@(CH_2_)_3_Im}C(CN)_3_ catalyst for 2-amino-3-cyanopyridine preparation *via* an anomeric based oxidation’ by Mohammad Ali Zolfigol *et al.*, *RSC Adv.*, 2016, **6**, 50100–50111, https://doi.org/10.1039/C6RA12299J.

The Royal Society of Chemistry hereby wholly retracts this *RSC Advances* article as the synthesis of {Fe_3_O_4_@SiO_2_@(CH_2_)_3_Im}C(CN)_3_ reported in the article, whereby tricyanomethane is used as a starting material, is not reproducible. The authors stated that they did not report the synthesis of tricyanomethane in the published paper as they purchased this compound from a commercial center and used it in the synthesis of ionic liquids, molten salts and various kinds of catalysts. The authors thought the reaction between tricyanomethane and organic bases is a simple acid–base reaction, therefore they did not cite the previously reported literature and its related history for the preparation of tricyanomethane. However, according to papers by Banert *et al.*,^1,2^ and based on their obtained analysis of the chemical sold to them as tricyanomethane, it became clear to the authors that this purchased compound was not tricyanomethane as there were differences in the ^1^H NMR and ^13^C NMR chemical shift between the purchased compound and the reports of Banert *et al.*^1^ According to these documents, the authors believe that the compound sold to them as tricyanomethane was fake. While the authors have now re-prepared {Fe_3_O_4_@SiO_2_@(CH_2_)_3_Im}C(CN)_3_, by synthesising potassium tricyanomethanide as a starting material for the synthesis,^3–5^ the synthesis reported in this article is not accurate. Therefore, this article is being retracted to avoid misleading readers and to protect the accuracy and integrity of the scientific record.

Mohammad Ali Zolfigol and Meysam Yarie oppose the retraction. Mahya Kiafar, Avat (Arman) Taherpour and Mahdi Saeidi-Rad were contacted but did not respond.

Signed: Laura Fisher, Executive Editor, *RSC Advances*

Date: 17^th^ August 2022

## Supplementary Material
